# Nonlinear effects of traffic statuses and road geometries on highway traffic accident severity: A machine learning approach

**DOI:** 10.1371/journal.pone.0314133

**Published:** 2024-11-22

**Authors:** Yao Liang, Hongxia Yuan, Zhenwu Wang, Zhongjin Wan, Tiantian Liu, Bing Wu, Shijie Chen, Xiaobo Tang

**Affiliations:** 1 Green and Low Carbon Transport Research Centre, Sichuan Communication Surveying and Design Institute Co., Ltd, Chengdu, China; 2 School of Transportation and Logistics, Southwest Jiaotong University, Chengdu, China; 3 Engineering and technology department, Sichuan Chengnan Expressway Co., Ltd, Chengdu, China; 4 Faculty of Geosciences and Engineering, Southwest Jiaotong University, Chengdu, China; Nanjing Forestry University, CHINA

## Abstract

The purpose of this study is to explore nonlinear and threshold effects of traffic statuses and road geometries, as well as their interactions, on traffic accident severity. In contrast to earlier research that primarily defined road alignment qualitatively as straight or curved, flat or slope, this study focused on the design elements of road geometry at accident locations. Additionally, this study considers the traffic conditions on the day of the accident, rather than the average annual traffic data as previous studies have done. To achieve this, we collected road design documents, traffic-related data, and 2023 accident data from the Suining section of the G42 Expressway in China. Using this dataset, we tested the classification performance of four machine learning models, including eXtreme Gradient Boosting, Gradient Boosted Decision Tree, Random Forest, and Light Gradient Boosting Machine. The optimal Random Forest model was employed to identify the key factors infulencing traffic accident severity, and the partial dependence plot was introduced to visualize the relationship between severity and various single and two-factor variables. The results indicate that the percentage of trucks, daily traffic volume, slope length, road grade, curvature, and curve length all exhibit significant nonlinear and threshold effects on accident severity. This reveals sepecific road and traffic features associated with varying levels of accident severity along the highway section examined in this study. The findings of this study will provide data-driven recommendations for highway design and daily safety management to reduce the severity of traffic accidents.

## 1. Introduction

In 2022, more than 256,409 road traffic accidents occurred in China, resulting in 60,679 deaths, 263,621 injuries, and direct property losses of 1,239.26 million RMB, according to data from the National Bureau of Statistics [1]. Road traffic accidents have become one of the leading causes of death. Due to the high speeds on highway, traffic accident can lead to more severe casualties and property losses [2]. In the field of highway traffic safety research, reducing the severity of traffic accidents has consistently been a major concern for researchers and practitioners.

Identifying the influencing factors of accident severity and understanding the relationships between them are crucial for formulating effective traffic accident prevention strategies. The influencing factors of road traffic accident severity are typically classified into driver, vehicle, road, and environment-related factors [3, 4], from which researchers identify the keys. Driver’s age [5, 6], unsafe driver behavior such as fatigue and drunk driving [5, 6], road-related factors like road geometry [5, 7–9] and road type [10, 11], traffic-related factors including annual average daily traffic [11–13], heavy vehicle percentage [11, 14], and speed limit [15], as well as inclement weather conditions [7, 11], have all been found to significantly impact accident severity. Although drivers play a significant role in traffic accidents, controlling driver behavior and vehicle performance on the highway can be challenging. Moreover, improving road conditions and adverse traffic statuses are helpful to indirectly control and avoid driver’s unsafe behavior and vehicles unsafe state, thus reducing the occurrence of traffic accidents [16]. For example, well-designed road alignment helps to prevent driver errors, reduce the difficulty of vehicle operation, and lower the likelihood of vehicle failure [17]. Implementing measures to alleviate adverse traffic conditions, such as congestion and the mixing of trucks and cars, can help mitigate drivers’ unsafe behaviors stemming from anxiety and impatience.

Examining the impacts of road design feature and traffic characteristics on the highway accident severity is crucial for improving highway design quality and preventing accidents. While many studies have examined these impacts in the past, most treat road alignment as categorical variables, defining them simply as straight or curved, flat or slope [7, 11, 16, 18–20]. This qualitative approach limits the ability to derive valuable insights for high-quality road design aimed at enhancing traffic safety. Moreover, existing studies primarily consider annual average traffic volume and truck percentages [2, 12, 13, 21], which may significantly differ from the conditions at the time of the accident. To address this gap, we collected road design documents and daily traffic data, and focus on the specific road geometry design element values at the location of each accident and the traffic conditions at the time of occurrence. This approach enables a more comprehensive assessment of how these factors contribute to varying levels of accident severity.

The nonlinear relationship between contribution factors and accident severity well established and has been extensively studied [5, 9, 22, 23]. The related studies primarily relied on statistical analysis methods, such as multinomial logistic regression model, logit model and its extensions [5, 11, 12, 14, 24, 25]. However, such statistical models are based on strong assumptions of log-linear [5, 13] or polynomial [23] relationships between independent and dependent variables, which may not hold true in many cases. As a result, they are insufficient to fully explore the complex nonlinear effects of contributing factors on accident severity. In recent years, with the development of machine learning algorithms and their modeling advantage of not requiring predefined relationships, Random Forest (RF), eXtreme Gradient Boosting (XGBoost), Gradient Boosted Decision Tree (GBDT), Light Gradient Boosting Machine (LightGBM) and other machine learning methods have been employed to explore the nonlinear effects on accident severity [5, 8, 22, 26]. Moreover, compared to traditional statistical models, machine learning methods have demonstrated superior classification and prediction performance [5, 9, 13].

The goal of this paper is to give an insight into the nonlinear and threshold effects of traffic status and road geometry and their interactions on highway traffic accident severity. Based on the 2023 imbalanced traffic accident data of the Suining section of the G42 Expressway in China, this paper proposes a data-driven framework. First, we select four classification models including RF, GBDT, XGBoost, and LightGBM to evaluate their performances in classifying the accident severity. The best-performing model, RF, is then employed to identify the key factors influencing traffic accident severity. Further, we introduce partial dependence plot to explain the RF model and explore the nonlinear rela-tionships between accident severity and individual or paired factors related to traffic status, road geometry. The findings of this study provide valuable insights for high-quality road design and traffic safety management.

The remaining sections of this paper are structured as follows. Section 2 reviews the relevant existing literature. Section 3 decribles the dataset and intrudece the methodology adopted in this paper. Section 4 discusses the model results. Section 5 summarizes the main findings and the future work.

## 2. Literature review

The analysis of factors influencing traffic accident severity has long been a subject of researchers’ attention. In practice, traffic accident severity is typically classified according to property damage, injuries, and fatalities [27]. Researches related to the influencing factors that contribute to traffic accidents severity have two main objectives: (1) to identify the crucial contributing factors and (2) to explore the influence mechanism between these factors and the accident severity. The summary of these researches is shown in Table 1.

**Table 1:** The summary of previous literatures related to accident severity.

Author	Goal			Influencing factors considered									Severity considered	Models
	A	B	C	I	II	III	IV	V	VI	VII	VIII	IX		
Sattar et al. [10]			√		√	√		√	√		√	√	severe, non-severe	GNN, RF, XGBoost, ANN
Yan et al. [8]	√	√				√		√	√	√	√	√	non-fatal, fatal	LightGBM
Mohammadpour et al. [28]	√		√		√	√		√	√		√	√	fatal and severe injury, less severe injury, PDO	RF, KNN, GBDT, SVM, Multi-Layer Perceptron
Hyodo et al. [11]	√	√		√		√	√	√	√	√			minor, severe, or fatal	Ordered Probit Model
Hosseinzadeh et al. [6]	√	√	√	√	√	√		√		√			fatal, non-fatal	SVM, random parameter logit model
Li et al. [5]	√	√	√	√		√		√	√	√		√	incapacitating crash, fatal crash	RF, GBDT, AdaBoost, Mixed Logit
Islam et al. [7]	√	√		√		√		√	√	√			fatal, non-fatal crashes	multinomial logit model
Ahmed et al. [18]	√	√	√	√		√		√	√		√	√	fatal, serious, minor, and non-injury	RF, Decision Jungle, AdaBoost, XGBoost, LightGBM, CatBoost
Shiran et al. [13]	√		√	√		√	√	√	√	√			PDO, fatality, severe injury, other visible injuries, and complaint of pain	MNL, ANN-MLP, CHAID, and C5.0
Zainuddin et al. [16]	√					√	√	√			√	√	non-fatal or fatal accident.	Descriptive and chi-square test
Ahmed et al. [18]	√	√	√	√		√		√	√		√	√	fatal, serious, minor, and non-injury crashes	RF, Decision Jungle, AdaBoost, XGBoost, LightGBM, CatBoost
Zhou et al. [29]	√	√	√	√	√	√		√			√		no injury, injury, fatality	MNL, Naive Bayes, SVM, and XGBoost
Panda et al. [30]	√	√	√		√	√		√		√			killed and injured	SVM, RF, GBDT, XGBoost
Yang et al. [22]	√	√				√		√	√				property loss, Injuries, Fatal	XGBoost + Bayesian network model
Mahashhash et al. [24]	√	√			√	√				√	√		non-severe injury, Severe injury or fatal	Binary logit model
Lee et al. [31]	√	√		√	√	√			√		√	√	PDO, bodily damage	Logit Model

A. Identify the key contributing factors; B. Examine the relationships between factors and accident severity; C. Compare several model’s performance.

I. Driver-related and driving behavior factors. II. Vehicle-related factors. III. Road geometry factors. IV. Traffic characteristic factors V. Weather factors. VI. Lighting conditions. VII. Crash characteristic factors. VIII. Temporal variables. IX. Other environment factors, such as built environment, spatial configuration.

Regarding the influencing factors, a survey conducted in Sistan and Baluchestan Province showed that human factors were the most important factor contributing to the increase in road traffic accidents, followed by vehicle status, road status, and environmental conditions [3]. Through a review of relevant existing literature, Ditcharoen concluded that the factor with the greatest impact on road traffic accident severity was vehicle speed, followed by human-related factors, including driver fatigue and alcohol consumption [4] The research in [5, 6] both analyzed the factors contributing to truck-related crashes, finding that driver fatigue was the significant factor leading to the severity of crashes. The latter also showed that enough width of curbs, medians, lanes and shoulders can prevent severe truck-related crashes. Eboli classified relevant influencing factors into three angles: road, external environment, and driver, and analyzed the factor characteristics influencing the severity of different crash types [20]. Zainuddin et.al specifically identified important factors leading to fatal heavy-goods vehicle (HGV) crash from the road and environment perspectives, finding that road geometry, shoulder type, road type, speed limit, and light conditions contributed to fatal crash, while the effects of road defects, road surface type, road surface condition, weather, month, and day of the week were not strong [16]. Hyodo found that in addition to traffic conditions and road-related factors, weather also had a significant impact on accident severity [11]. I. M. Almadi focused on investigating the impact of changes in speed limits under weather conditions on vehicle crashes on highway, indicating that crashes mainly occurred in snowy and icy weather conditions, and in these adverse weather conditions, driving speed had a significant impact on the occurrence of traffic accidents [15].

To examine the relationships between influencing factors and accident severity, numerous researchers have constructed various models based on actual accident data, among which statistical models are the most common and dominant ones, such as multinomial logistic regression model [7, 13, 15], ordered logit model [11, 12], binary logit model [24], and random parameter logit model [5, 14, 25, 32]. Haghighi et.al established a multilevel ordered logit model to quantify the impacts of geometric features and environmental conditions on accident severity, finding that 10-foot-wide lanes and narrower shoulders were significantly associated with accident severity, while increasing driveway density and barrier length could reduce accident severity [12]. Lee et al. used a logit model to specifically investigate how the age and gender of negligent drivers influence crash severity [31]. Results showed that as age increases, the probability of drivers suffering physical injuries or fatalities decreases, but this trend is weak before old age. To investigated the impact of weather on road vehicle collision severity, Islam et al. employed a multinomial logistic regression model and discovered that increases in humidity, temperature, and rainfall all increased the probability of fatal collision accidents, while wind speed had no significantly impact [7]. Hyodo et al. indicated that temperature and visibility factors might increase the likelihood of severe and fatal multi-vehicle accidents in a research by using ordered logit model [11]. A mixed logit model is used by Milton et al. to examine the influences of traffic, road, and weather [14]. Results showed that an increase in average daily traffic per lane would decrease the probability of property-damage-only accidents, while an increase in average daily truck traffic would decrease the probability of injury accidents, and an increase in the percentage of trucks might slightly increase the occurrence of possible injury accidents. Other researches showed that head-on collision, elevated speed, the use of private car, and weekend also significantly caused the severe injuries [24]. However, most statistical models have their own model assumptions and predefined relationships between independent and dependent variables, especially log-linear relationships, which is not flexible enough to capture the actual complex nonlinear relationships [5, 13, 24].

Compared with traditional statistical models, machine learning methods, such as XGBoost, RF, GBDT, and Support Vector Machines (SVM), have been widely used to uncover and examine nonlinear relationships between independent and dependent variables, as they do not require predefined relationships between them [33]. Techniques like partial dependence plot (PDP) and SHAP (SHapley Additive exPlanation) value are often used to visualize these relationships. Li et al. compared machine learning models such as GBDT and RF, with traditional mixed logit model and demonstrated that machine learning models, especially GBDT, can effectively identify key influencing factors of large truck crashes, and can reveal the nonlinear relationships between them by partial dependence plots [5]. Yang et al. identified that built environment factors, particularly demographics, land use, and road networks, are highly correlated with three injury types of truck-related crashes, and nonlinear relationships between them were exist [9]. While Yang et al. discovered nonlinear interactions between various factors in the road and environment dimensions by using XGBoost and SHAP method [22]. The studies utilizing machine learning models all demonstrated the existence of nonlinear relationships between influencing factors and accident severity. However, existing applications of machine learning method in accident severity analysis have primarily focused on the classification prediction of accident severity [10, 28, 29, 34], emphasizing the predictive performance of varies machine learning models versus traditional statistical models [19, 35]. Researches specifically employing machine learning models to explore the nonlinear effects of independent variables on the dependent variable remain relatively scarce.

Furthermore, performance comparisons of various classification models reveal that no single machine learning model consistently outperforms others under different research scenarios and accident datasets. In a study identifying risk levels of highway bridge segments, Zhao et al. found that Random Forests had better predictive accuracy than traditional multinomial logistic regression [26]. Zhou et al. employed five classification models, including a multinomial logistic regression model, to investigate the influencing factors of injury severity for passenger car and truck drivers, finding that XGBoost performed better in terms of G-mean, overall accuracy, and area under the curve [29]. Ahmed et al. utilized six explainable machine learning models, mainly including Random Forest, XGBoost, CatBoost, and LightGBM, to identify contributing factors of road accident [18]. They found that Random Forest achieved the highest prediction accuracy, precision, and recall under the balance-addressed accident severity data, which is consistent with the result reported in Mohammadpour et al. [28]. The later also indicated that GBDT performed better under the imbalanced data.

**Fig 1:**
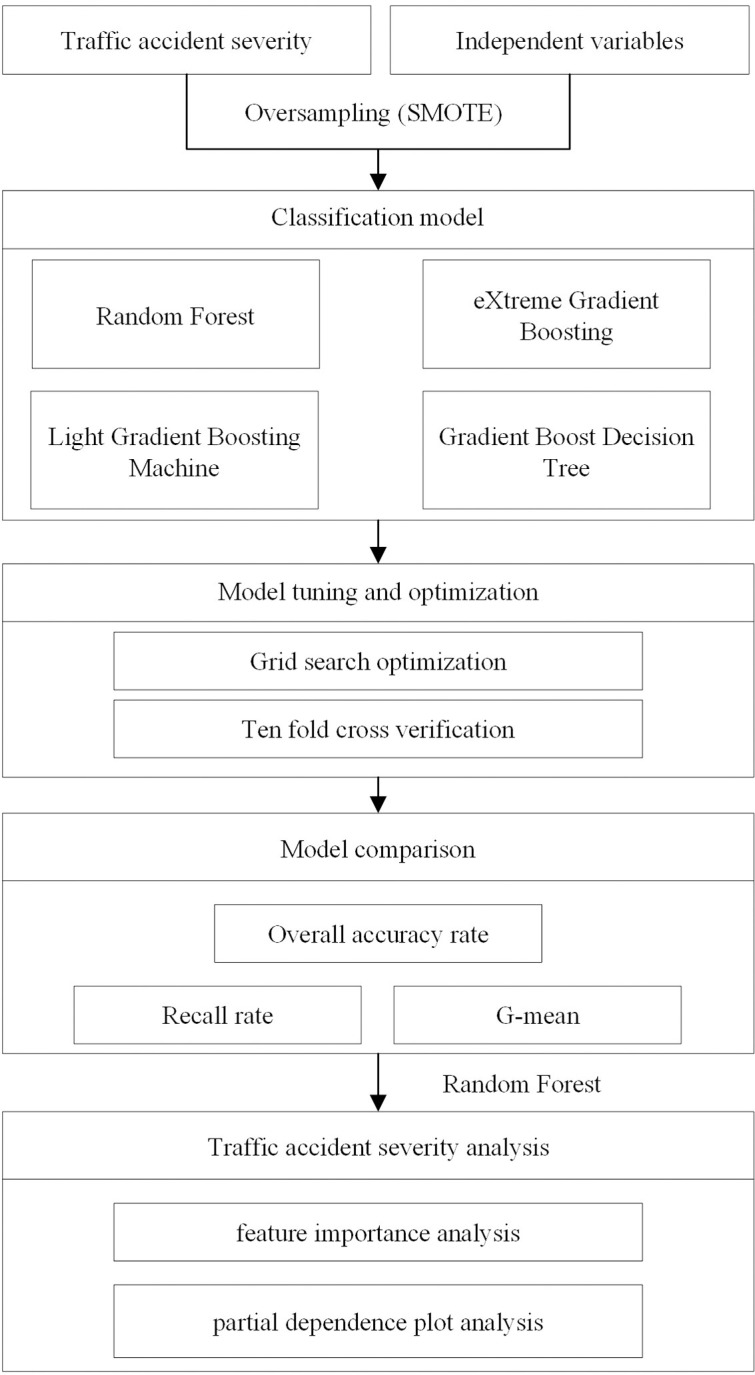
Framework of modeling and accident severity analysis.

Therefore, to identify a suitable machine learning method for this study’ dataset, we selected four machine learning models, including RF, GBDT, XGBoost, and LightGBM, each recognized for its effective classification performance in accident analysis research. We evaluated each algorithm’s classification capabilities using several performance metrics such as prediction accuracy, recall, and G-mean. The model with the highest performance was ultimately chosen to explore nonlinear relationships. The research framework is shown in Fig 1.

## 3. Materials and methods

### 3.1 Data description and varibales selection

The Suining section (milepost range K1819-K1912) of the G42 Expressway in China spans 94 km and serves as a crucial highway connecting Chengdu, the provincial capital of Sichuan, with the major cities of Suining and Nanchong. This section experiences a high average daily traffic volume, particularly during holidays, frequently leading to congestion. Historical traffic accident statistics from 2023 shows an average of 11 accidents per kilometer per year on this section, significantly higher than the adjacent section’ rate of 6. The Suining section is a fully access-controlled, four-lane, bidirectional highway in a hilly area, characterized by relatively complex road conditions. It is designed for a speed of 100 km/h, with a carriageway width of 3.75 m, a median greenbelt width of 3.5 m, and a shoulder width of 3.75 m. To investigate the impact of traffic conditions and road geometry features on accident severity, we collected traffic accident data for the entire year of 2023, along with road design documents and traffic-related data from the highway operator.

#### (1) Traffic accident data

The accident data was obtained through the highway operator and originally collected from traffic accident records complied by the Sichuan Provincial Traffic Police Corps Highway Detachment during each accident investigation. The data obtained in this paper primarily include information on the date and time of the accident, location, weather conditions, type of accident, and details regarding casualties and road property damage. In the one-year period, a total of 968 traffic accidents occurred on the selected highway section. This included 54 accidents resulting in minor injuries, 5 accidents leading to serious injuries or fatalities, 280 accidents involving only road property damage, and 629 minor accidents that did not involve casualties or road property damage.

Researchers typically categorize accidents into three types: property damage only, injury accidents, and fatal accidents [13, 18, 22, 29]. In some studies, due to the low incidence of serious injuries and fatalities, these accidents are reclassified together with minor injury accidents as bodily damage accidents [31, 36], which also applies to the accident data in this study. Accidents involving property damage only are the most common type, which can be further subdivided into road-related and non-road-related damages. The former includes damage to roads and associated facilities, such as guardrails, automated toll barriers, and asphalt surfaces, while the latter primarily involve vehicles and the cargo. Road-related property damage accidents often imply that vehicles may drive out of the road, and if safety barriers are not in place, this could lead to more severe traffic incidents [6, 11]. Therefore, it is essential to differentiate between road property damage and non-road property damage accidents to conduct an in-depth analysis of how various factors influence the severity of accidents. Considering the characteristics of accidents, this study ultimately classifies accident severity into three categories: (1) no injury and no road property damage (referred to as NINP), (2) no injury but with road property damage only (referred to as NIWP), and (3) with injuries and fatalities (referred to as WIWF), accounting for 64.97%, 28.93%, and 6.10%, respectively.

#### (2) Road geometry factors

Unlike most previous research that qualitatively defines road alignment as straight or curved, flat or sloped, or combined alignment types [7, 11, 16, 18], this study additionally selects horizontal and vertical alignment design elements based on engineering design experience, past research, and exploratory analysis. Key elements affecting road traffic safety primarily include straight length, curve length, curvature, superelevation, road grade, and slope length [17, 21, 37]. To obtain the corresponding alignment element values at each accident location, we segment the road both horizontally and vertically. Horizontally, we divide the road into tangent and curve segments based on curvature [17, 23], with a curvature of 0 for tangent segments. The length of each horizontal segment is defined as either straight length or curve length. It is important to note that the curve superelevation is equivalent to the cross slope on the tangent segment. Vertically, we segment the road based on changes in gradient, with points where the gradient changes serving as the starting or ending points of a segment, indicating that the gradient within the same vertical segment is uniform. For accidents occurring on flat segments, both the road grade and slope length are set to 0. Moreover, referring to the Specifications for Highway Safety Audit [38], we classify road alignment combinations into four types based on a curve radius threshold of 1000 m and a gradient threshold of 3%: straight + flat, straight + slope, curve + flat, and curve + slope.

#### (3) Traffic conditions

The traffic data we collected consists of daily vehicle counts by type between adjacent toll stations on the expressway. Each direction along the study highway section contains eight toll stations, forming seven toll units. The daily traffic volume and truck percentages within the same toll unit vary from day to day. In this study, each individual accident serves as the unit of analysis. We identify the toll unit for each accident based on its location and use the traffic volume and percentage of trucks on the day of the accident within that toll unit as traffic related factors. The truck percentage is calculated as the ratio of truck traffic volume to total traffic volume on that day. Both factors are standardized to passenger car units (pcu) using appropriate conversion coefficient to account for different vehicle types.

#### (4) Other environment factors

In addition to traffic conditions, we consider other environmental factors such as weather, lighting, and day type that may influence traffic accidents, all of which are commonly examined in most literature [7, 26, 29]. The traffic accident data we collected records weather conditions, which we categorize as sunny, overcast, light to moderate rain, and heavy to torrential rain [18, 26]. Lighting conditions are classified into two categories: daytime and nighttime. Daytime is defined based on month and time of day, specifically from 6:00 AM to 8:00 PM during April to September, and from 7:00 AM to 7:00 PM from October to March of the following year, with all remaining hours classified as nighttime [11, 39].

The description of all variables used in this study is shown in Table 2. Categorical variables are coded and continuous variables are calculated in actual value. It is important to note that 650 accidents occurred on curve segments, while 318 occurred on tangent segments, and no accidents took place on segments with a 0% road grade.

**Table 2:** Description of the independent and dependent variables.

Variable types	Variables	levels	Code	Count	Percent
Traffic accident severity		NINP	0	629	64.97%
		NIWP	1	280	28.93%
		WIWF	2	59	6.10%
Environment conditions	Weather conditions	Sunny	1	613	63.33%
		Overcast	2	132	13.64%
		Light rain	3	168	17.36
		Heavy rain	4	55	5.68%
	Lighting condition	Daytime	1	715	73.86%
		Nighttime	2	253	26.14%
	Day type	Weekdays	1	608	62.81%
		Weekends	2	187	19.36%
		Holidays	3	173	17.87%%
Road geometry	Combine alignment	Straight + flat	1	767	79.24%
		Curve + flat	2	187	19.32%
		Straight + slope	3	14	1.45%
	Horizontal alignment	Straight	1	318	32.85%
		Left curve	2	359	37.09%
		Right curve	3	291	30.06%
**Variable types**	**Variables**	**levels**	**Min**	**Max**	**Mean**
Road geometry	Tangent length ^a^ (m)	Continuous variable	245.75	2413.08	916.56
	Curve length ^b^ (m)		514.38	3140.25	1044.47
	Curvature ^c^ (*0.001)		0	2	0.5024
	Superelevation (%)		0.13	8	2.35
	Road grade (%)		-3	3	0
	Slope length (m)		320	2200	1068.10
Traffic status	Traffic volume (1000 pcu/day)		7.25	51.53	28.38
	Percentage of trucks (%)		1	37	19.81

a. 0 is not considered here, which represents that the horizontal segment is curve. b. 0 is not considered here, which represents that the horizontal segment is tangent. c. If without considering the tangent segment (curvature = 0), the minimum curvature is 0.167, and the mean is 0.7482.

### 3.2 Data imbalance treatment

As shown in Table 2, there are 629 NINP accidents, 280 NIWP accidents, and 59 WIWF accidents, showing a distinctly unbalanced characteristic, which is common in many multi-classification datasets [28, 29, 36]. To improve the classification performance of a machine learning model, it is necessary to increase the number of samples for the minority classes (i.e. oversampling) to balance the proportion among different classes in the dataset. SMOTE (Synthetic Minority Over-sampling Technique), a data enhancement technique for data balancing, was first proposed by Chawla et al. [40] and subsequently widely applied in imbalanced data processing [29]. The core idea of SMOTE is to increase the number of samples in the minority class by synthesizing new samples, thereby achieving a more balanced distribution classes in the dataset [8]. This approach is particularly suitable for the imbalanced accident data in this paper, as the percentage of WIWF incidents is very small. The basic steps of SMOTE are as follows:

1. Select K nearest neighbors: For a minority class sample, first select K nearest neighbors in a minority class sample. K is a pre-set hyperparameter that controls the number of new samples synthesized.
2. Random generation of new samples: For each minority class of samples, a sample is randomly selected from its K nearest neighbors, and the difference between the two samples (the difference in position in the feature space) is calculated.
3. Synthesize a new sample: For each difference, multiply by a random number (usually a random number between [0,1]), and then add the result to the original sample to get a synthesized new sample.
4. Repeat steps: Repeat the above steps until a predetermined number of new samples are generated.
5. After the oversampling process, the data are prepared for the following works.

### 3.3 Random Forest (RF)

RF, introduced by Breiman [41], is an ensemble learning method that constructs multiple decision trees during training and outputs the mode of the classes (for classification) or mean prediction (for regression) of the individual trees. Each tree is built using a random subset of features and data samples, which helps reduce overfitting and increase generalization. For classification problem, RF decides the final classification by majority voting. That is, the final classification result $\hat{y}$ for the sample is the mode of the prediction result of each decision tree:

$$\hat{y}=\mathrm{mode}(y_{1},y_{2},\cdots ,y_{T})$$ (1)

Where *y*_*t*_ is the prediction from the t-th tree, and *T* is the total number of trees. In the implementation of RF, two key parameters must be determined: the total number of trees and the number of features randomly selected as candidates for each node split. RF can provide feature importance metrics [42], based which we can identify the key factor contributing accident severity.

### 3.4 Gradient Boosted Decision Tree (GBDT)

The GBDT is an ensemble method that builds trees sequentially. Each tree is trained to predict the residuals (errors) of the previous trees, effectively minimizing the loss function through gradient descent [43]. This allows the model to correct its errors iteratively. The iterative update rules are as follows:

$$F_{m}(x)=F_{m-1}(x)+\eta \cdot h_{m}(x)$$ (2)

Where *x* denotes the set of dependent variables, *F*_*m*_(*x*) is the outcome at iteration *m*, *h*_*m*_(*x*) is the base learner (decision tree), and *η* s the learning rate. GBDT is flexible and performs well on various tasks but prone to overfitting, particularly if hyperparameter tuning is not conducted effectively. To mitigate this risk, tree complexity and learning rate serve as regularization parameters that need careful adjustment. The learning rate specifically controls the pace of updates following each iteration, playing a crucial role in stabilizing the model’s performance.

### 3.5 eXtreme Gradient Boosting (XGBoost)

XGBoost is an optimized implementation of GBDT that incorporates regularization to prevent overfitting and speed up computations [44]. It also supports parallel processing, which increases training efficiency. The goal of XGBoost is to minimize the following loss functions:

$$L(\theta )=\sum_{i=1}^{n}l(y_{i},{\hat{y}}_{i})+\sum_{k=1}^{K}\Omega (f_{k})$$ (3)

where *θ* denotes model parameters which need to be careful tuning; *y*_*i*_ and ${\hat{y}}_{i}$ is the actual label and model prediction for the *i*_*th*_ data sample, respectively; *l* is the loss function of the *i*_*th*_ data sample; Ω(*f*_*k*_) is the regularization term, aiming to control the model complexity to aviod overfitting; *K* is the number of trees.

### 3.6 Light Gradient Boosting Machine

LightGBM is a gradient boosting framework developed for efficiency and scalability, especially with large datasets. It uses a histogram-based approach for finding the best split points and supports categorical features directly, reducing preprocessing time. The objective function of LightGBM is similar with that of XGBoost. However, LightGBM employs a leaf-wise growth strategy for trees, directly handles categorical features, and is optimized for faster training on large datasets. In contrast, XGBoost adopts a layer-wise tree growth approach and requires feature encoding for categorical variables [18]. Compared with other machine learning models, LightGBM excels with large datasets and low memory usage but may overfit on smaller datasets and needs parameter tuning to manage this risk.

### 3.7 Perfermance metrics

Accuracy, recall, and precision are critical metrics for evaluating machine learning models, providing insights into different aspects of model performance. Accuracy serves as a general and intuitive indicator of correct predictions [8, 10, 13, 18]. However, for the imbalanced datasets, relying solely on overall accuracy can lead to skewed evaluations. Tharwat et al. demonstrated that both recall and precision are valuable for evaluating classification performance with data imbalances [45]. Recall emphasizes the model’s capability to correctly identify true positive samples among all predicted true samples, which is crucial in scenarios where missing a positive case is costly, while precision quantifies the proportion of predicted positive samples that are actual positive. Given that this study aims to accurately classify the categories of accident severity, focusing on the recall for each category aligns more closely with this objective. Moreover, the geometric mean (G-mean) is also a widely used metric in imbalanced dataset analysis [24, 28, 29], which combines both sensitivity and specificity, providing a balanced view of performance [45]. Based on this analysis, overall accuracy, recall, and G-mean have been selected to ensure a comprehensive evaluation of model performance in this study. For three-classification model, the relevant definitions of the metrics are as follows:

$$Accuracy=\sum_{i\in K}TP_{i}/\sum_{i\in K}\left(TP_{i}+FN_{i}\right)$$ (4)

$$Recall_{i}=TP_{i}/\left(TP_{i}+FN_{i}\right)$$ (5)

$$G-mean=\sqrt[{3}]{Recall_{0}\cdot Recall_{1}\cdot Recall_{2}}$$ (6)

Where *i* indicates the class of traffic accident severity. *K* is the set of traffic accident severity classes (*K* = {0,1,2}). *TP*_*i*_, namely true positive, is the number of samples that are truly class *i* and predicted to be class *i*. *FN*_*i*_, namely false negative, is the number of samples that are truly class *i* but predicted to be non-class *i*. To calculate these metrics, the confusion matrix is calculated to identify *TP*_*i*_ and *FN*_*i*_. For three-classes accident severity, the confusion matrix is represented by a 3×3 table.

### 3.8 Partial dependence plot

Given the ability of partial dependence plot (PDP) to analyze the effects of single or multiple variables on the prediction results [26], we adopt PDP to visualize the model results to explore the nonlinear and interactive relationship between factors and traffic accident severity. If PDP needs to deal with multiple classes, it will plot per OvR (One vs Rest) class to show the effects of feature variables (explanatory variables) on each class. The partial dependence function is defined as follows:

$${\hat{f}}_{x_{S}}(x_{S})=E_{x_{C}}[\hat{f}(x_{S},x_{C})]=\int \hat{f}(x_{S},x_{C})dP(x_{C})$$ (7)

Where *S* is the set of features we are interested in, usually includes one or two features. *C* is the set of other features used in the classification model. *S* and *C* form all the feature sets of the model. *x*_*S*_ and *x*_*c*_ are eigenvectors corresponding to sets *S* and *C*, respectively, with the former used to plot the partial dependency functions.

The partial dependence function ${\hat{f}}_{x_{S}}$ is estimated by calculating the mean value in the model training dataset, see in (8), also known as the Monte Carlo method.

$${\hat{f}}_{x_{S}}(x_{S})=\frac{1}{n}\sum_{i=1}^{n}\hat{f}(x_{S},{x_{C}}^{\left(i\right)})$$ (8)

Where *x*_*C*_^(*i*)^ is the actual eigenvalue of the feature we are not interested in, and *n* is the number of samples in the dataset.

## 4. Results and discussion

In this study, open-source libraries like scikit-learn and PDPbox were employed for training machine learning models and drawing partial dependent plots. For model tuning and optimization, the dataset is randomly divided into training set and test set according to the ratio of 7:3. We used a grid search optimization method for hyperparameter tuning, accompanied by ten-fold cross-validation. These processes involved randomly splitting the training data into 10 subsets, where each training iteration utilized 9 subsets for training and 1 subset for validation. These works were implemented using the python programming language on the PyCharm platform. The experimental environment was Windows 11, 12th Gen Intel(R) Core (TM) i5-12500H 2.50 GHz with 16.0 GB RAM.

### 4.1 Classification performance comparation

**Fig 2:**
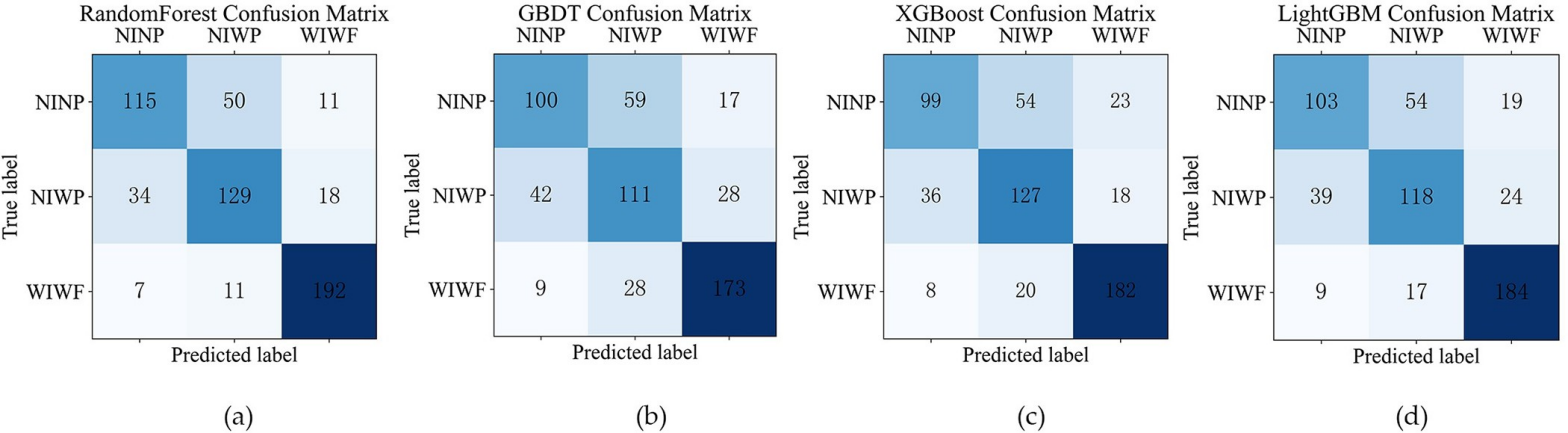
Confusion matrices for the four models.

The confusion matrixes obtained from the four models is shown in Fig 2, form which true positive (TP) and false negative (FN) of each severity class are identified to calculate the performance metrics. Based on the confusion matrixes, the overall accuracy, class recall, and G-mean are calculated, as shown in Table 3. The result shows that RF model achieves the highest accuracy of 76.90%, the highest G-mean of 75.23%, and highest recall rates of 65.34% for NINP, 71.23% for NIWP, and 91.43% for WIWF. This model demonstrates the best classification performance in this paper, especially in identifying injury and fatal accidents. Therefore, the RF model is employed to identify the significant contributing factors and examine the nonlinear effects.

**Table 3:** Classification performances of four models.

Performance metric	RF	GBDT	XGBoost	LightGBM
**Overall accuracy (%)**	76.90%	67.72%	71.96%	69.66%
**Class NINP recall (%)**	65.34%	56.82%	56.25%	58.52%
**Class NIWP recall (%)**	71.27%	61.33%	70.17%	65.19%
**Class WIWF recall (%)**	91.43%	82.38%	86.67%	87.62%
**G-mean (%)**	75.23%	65.97%	69.94%	69.40%

### 4.2 Feature importance ranking

**Fig 3:**
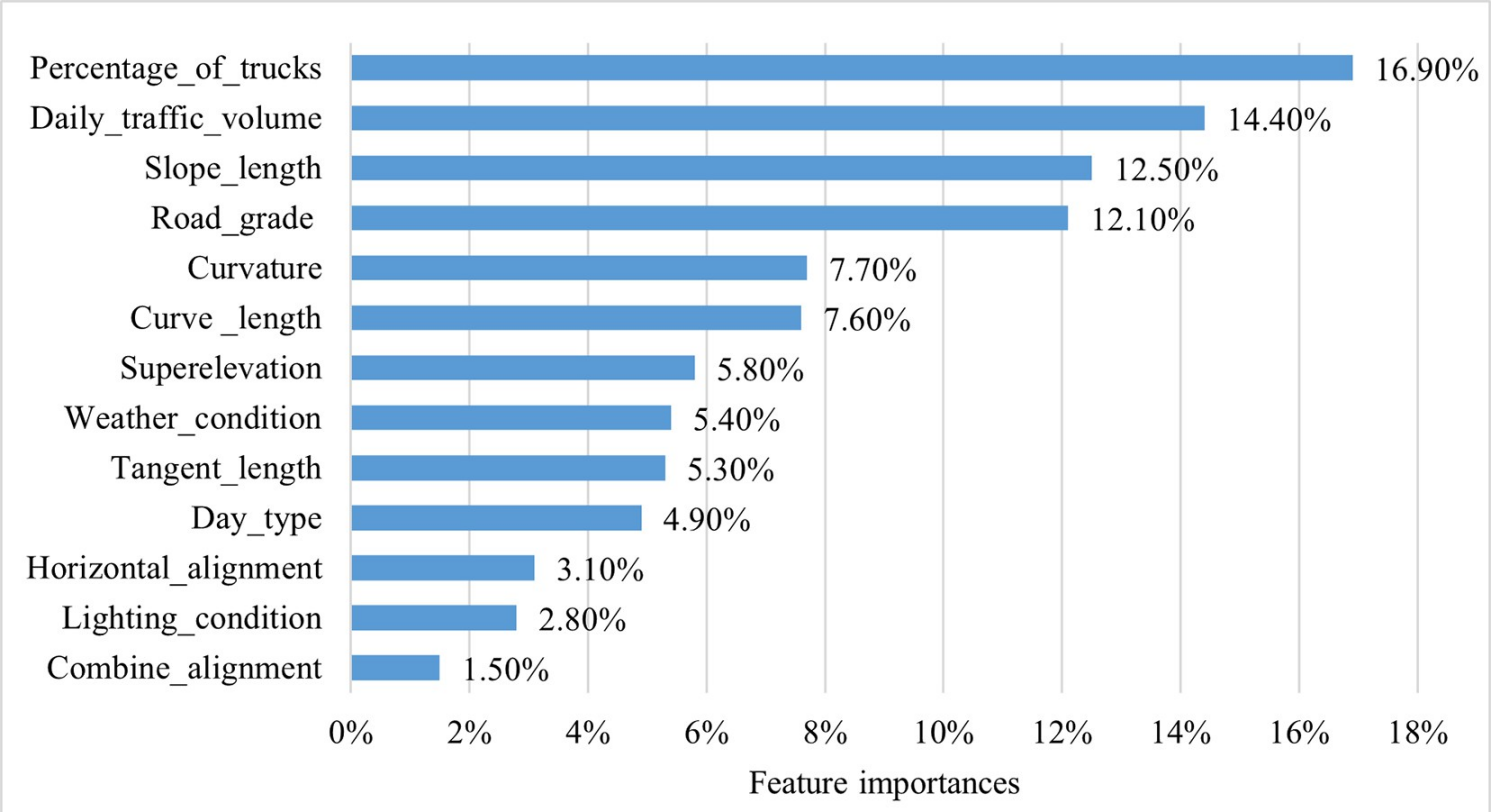
Feature importance of RF model.

By analyzing the contribution of each feature (explanatory variable) to RF’s classification performance, the importance of all features can be ranked. The higher the feature importance, the greater its impact on the classification results. As shown in Fig 3, the top six ranked variables are the percentage of trucks, daily traffic volume, slope length, road grade, curvature, curve length, with the importance values of 16.9%, 14.4%, 12.5%, 12.1%, 7.7%, and 7.6%, respectively. This indicates that traffic statuses and road geometry characteristics have a significant impact on traffic accident severity.

### 4.3 The nonlinear effect of a single factor

We used partial dependence plot (PDP) to more intuitively explore the nonlinear relationship between factors and traffic accident severity. In the PDP plot of a single feature for a class, the value of the ordinate represents the relative probability of the class prediction corresponding to the feature value. An ordinate value greater than 0 indicates that the feature value increases the probability of being predicted for the class; otherwise, it decreases the probability. If the ordinate value is close to 0, this feature value has no significant effect on this class. Base on the feature importance ranking in Fig 3, six feature variables with greater influence were selected to draw single-factor PDPs to examine their nonlinear effects. PDPs for other factors are provided in the Supporting Information section of this paper (See S2 File).

### Percentage of trucks

**Fig 4:**
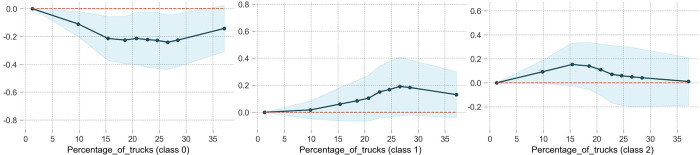
The partial dependence plot of the percentage of trucks.

Fig 4 shows the partial dependence plots of the percentage of trucks, in which class0, class1, and class2 represent NINP, NIWP, and WIWF, respectively. As depicted in Fig 4, percentage of trucks has significant nonlinear effect on accident severity. The ordinate value in the PDP for Class0 is less than 0, while it is greater than 0 for class1 and class2, indicating that mixed traffic of trucks and cars on the highway is associated to a lower likelihood of NINP accidents but a higher likelihood of NIWP and WIWF accidents. In addition, as the percentage of trucks increases, the probabilities of NIWP and WIWF accident rise. Once the percentage of trucks exceeds 20%, the probability of WIWF accident decreases, while the probability of NINF accident continue to rise until the percentage reaches 27%. This result may be attributed to the speed difference between trucks and cars. At lower truck percentages, traffic conditions are less complex but the average speeds are relatively high, leading to a higher likelihood of driving out of roadway and severe casualties. Once the truck percentage increases to a certain threshold, vehicle speeds decrease, potentially reducing the occurrence of casualties.

### Daily traffic volume

**Fig 5:**
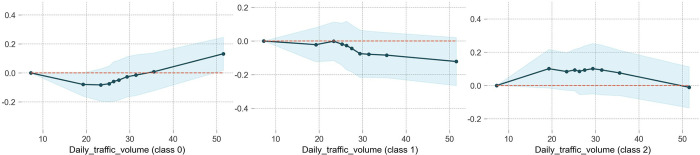
The partial dependence plot of daily traffic volume.

As observed in Fig 5, the effect of daily traffic volume on the severity of traffic accidents is nonlinear. When the traffic volume is below 20,000 pcu/day, an increase in traffic volume raises the probability of WIWF (class2) accident, and the probability remains high in the range of 20,000 to 32,000 pcu/day. This may be attributed to low traffic volumes creating a free traffic flow environment which encourages drivers to speed and become less attentive. This lack of vigilance makes them more prone to unsafe behaviors such as speeding and reckless lane changes, ultimately leading to accidents and injuries. The traffic volume has a completely opposite nonlinear effect on NINP (class0) accident, with 32,000 pcu/day serving as the threshold. For the NIWP (class1) accidents, a low traffic volume (below 25,000 pcu/day) has no significant effect, but once the volume exceeds 25,000 pcu/day, the likelihood of NIWP accidents decreases as traffic volume increases. This can be explained by the fact that as traffic volume grows, vehicles tend to travel at lower speeds and in queues, reducing the chances of vehicles running off the roadway into guardrails and increasing the likelihood of rear-end or side-swipe collisions.

### Slope length

**Fig 6:**
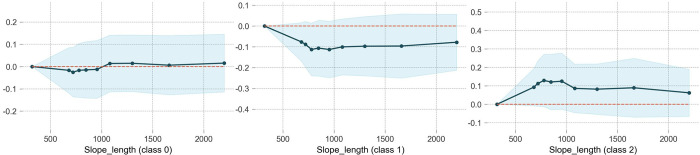
The partial dependence plot of slope length.

Fig 6 shows a threshold effect of slope length on all accident severity types, although the effct on NINP accident (class 0) is not siginificant. NIWP (class1) accident are less likely to occur on slope sections as the ordinate value less than 0 in PDF for class1. Similarly, Regardless of slope length, WIWF accident are more likely to occur on the slope sections, with a higher probability observed within the slope length range of 750 to 1000 m. Beyond this range, the likelihood decreases but still maintains high. This decline is likely due to the presence of traffic signs providing safety warnings on excessively long uphill or downhill sections, which promptes the driver to take preventive measures in advance.

### Road grade

**Fig 7:**
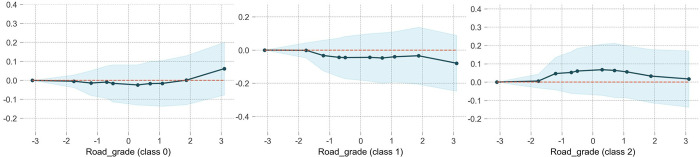
The partial dependence plot of road grade.

Fig 7 illustrates the impact of road grade on accident severity. Compared to a slope of -3%, the ordinate value for WIWF (class 1) is greater than 0, with significant values observed within the range of -1% to 1%. This suggests that accidents involving casualties are more likely to occur on relatively flat road segment. This may be attributed to the fact that drivers tend to exercise more caution when navigating slopes. Therefore, it is also important to implement safety driving warnings on flat road sections. In contrast, both NIWP and NINP accidents are less likely to occur on relatively flat road segment. NINP accidents are more common on uphill segments with gradient greater than 2%, while NIWP accidents are even less likely to occur on such segments, which aligns with expected outcomes.

### Curvature

**Fig 8:**
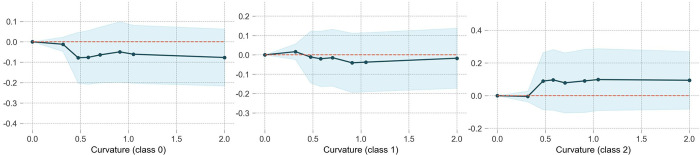
The partial dependence plot of curvature.

As shown an Fig 8, compared to curved segments with a curvature of less than 0.0005, WIWF (class2) accidents are more likely to occur on curved segments with a curvature greater than 0.0005 (i.e., radius lower than 2000 m), which starkly contrasts with NINP (class0) accidents. For NIWP (class1) accidents, the curvature has a minimal impact on occurrence. The likelihood of NIWP slightly decreases on curve segments with a radius smaller than 2,000 m. This demonstrates that smaller curve radius have a significantly adverse effect on traffic safety.

### Curve length

**Fig 9:**
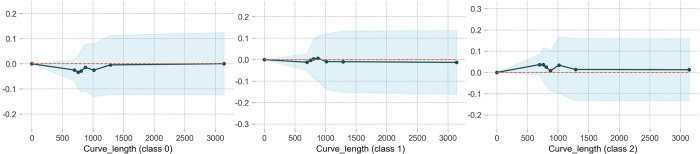
The partial dependence plot of curve length.

Fig 9 illustrates a limited impact of curve length on traffic accident severity. Simillar to curvature, the ordinate value of class2 in the PDP is greater than 0, meaning that WIWF accident is more likely to occur in the curve sgement compared to the straight segment, which contrasts with NINP (class0) accidents. The range of 750 to 1250 m is an threshold, where WIWF accidents are most likely to happen, while NINP accident is least likely to occure. Beyond the range, the effect of curve length on WIWF and NINP accidents remain almost unchanged.

### 4.4 The interaction effect under two factors

**Fig 10:**
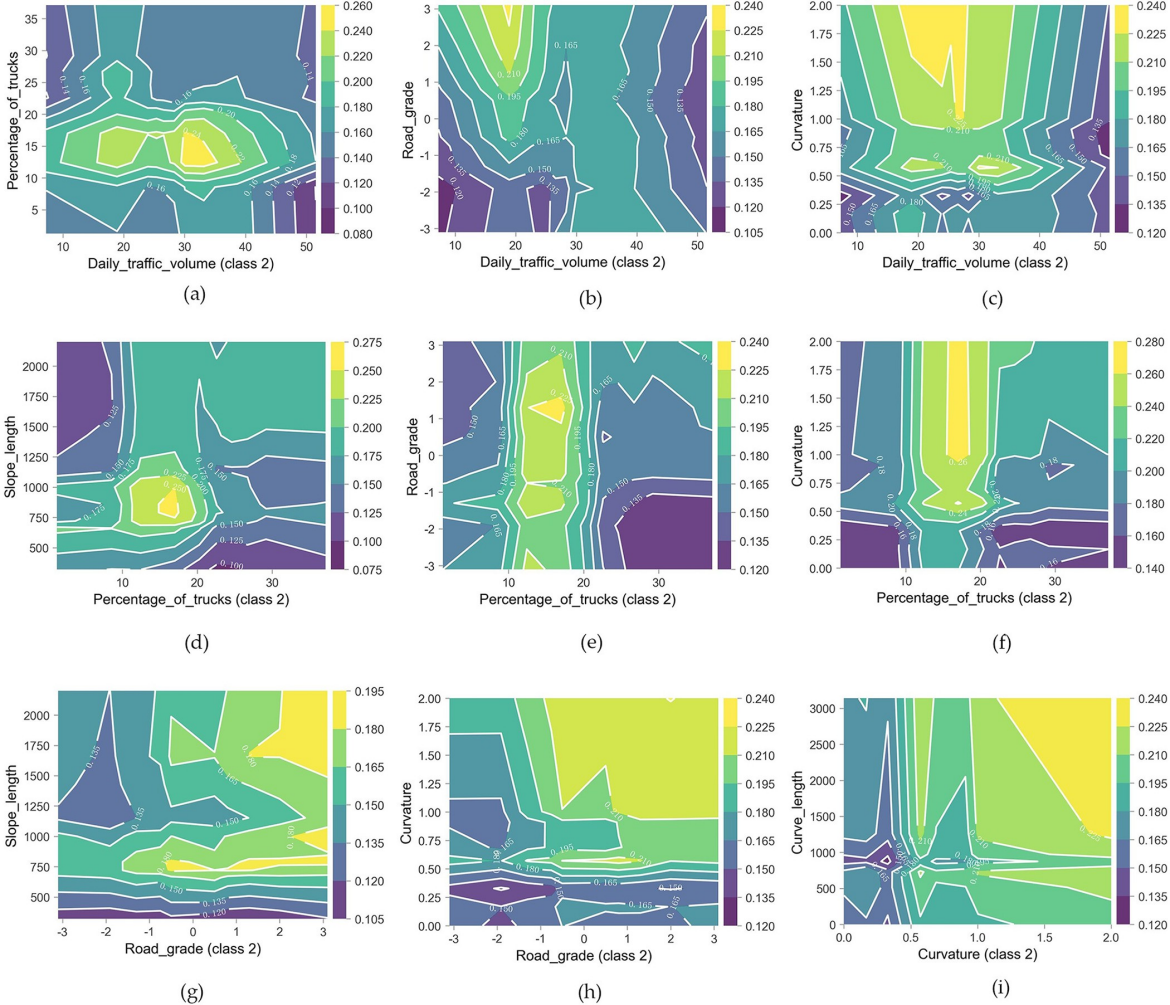
The partial dependence plots for WIWF accident under two interactive factors.

The causes of traffic accidents are complex and may be influenced by multiple factors. Since the accidents with injuries and fatalities (WIWF accidents) may bring serious casualties and property losses, this section focuses on how dual factors interactively impact the occurrence of WIWF accidents and constructs two-factor PDPs for analysis. The factors are selected from the top six most important features in Fig 3, which are related to traffic status and road geometry. Their interaction effects on accident severity are shown in Fig 10. In this figure, the horizontal and vertical axes represent the values of the main effect and interaction effect variables, respectively, and the vertical bar legend on the right displays the probability of WIWF accident as predicted by the RF model.

Fig 10(A) indicates that a truck proportion of 12% to 17% has a significant negative impact on different levels of daily traffic volume, particularly when the traffic volume is between 30,000 and 35,000 pcu/day. Fig 10(B) shows that the likelihood of casualty accident is highest on uphill sections when traffic volume is about 20,000 pcu/day. Fig 10(C) demonstrates that casualty accidents are more likely to occur on tangent or curve segments with larger radius when the traffic volume is between 25,000 and 30,000 pcu/day. Fig 10(D)–10(F) illustrate that the threshold effects of truck proportions on casualty accidents vary across road segments with different grades, slope lengths, and curvatures. Under mixed traffic conditions with a percentage of trucks ranging from 10% to 20%, special attention should be given to uphill slopes with lengths of 700 to 1,000 meters and curved segment with a radius of less than 2,000 meters (i.e., curvature greater than 0.0005). Interestingly, when the percentage of trucks exceeds 20%, casualty accidents are more likely to occur on uphill segments. Conversely, when the truck proportion is below 10%, there is a greater probability of casualties occur ring on downhill sections compared to uphill ones. These findings highlight the importance of considering both traffic conditions and road characteristics when assessing traffic safety risks.

Fig 10(H) and 10(I) illustrate the interaction effects of main road geometry combinations on fatal accidents. It is evident that the likelihood of casualty accidents is higher on relatively flat segments approximately 750 m in length, uphill sections longer than 1,250 m, curve segments with a radius less than 1,000 m and a length greater than 1,000 m, as well as combinations of non-downhill and curve with a radius less than 1,000 m. Therefore, these specific geometry features should be avoided in road planning and design, and daily operations should prioritize enhancing safety management for these segments, especially the curve and slope combinations and the small-radius curve.

## 5. Conclusions

Based on the 2023 traffic accident data, road design documents, traffic-related data of the Suining section of G42 Expressway in China, this paper investigates the complex nonlinear effects of traffic status, road geometry, weather, lighting conditions and day type on traffic accident severity by using Random Forest method and partial dependence plot. The main findings of this study are reflected in the following aspects.

1. We used the Random Forest model to evaluate the relative importance of 14 feature factors influencing severity of traffic accidents. Our analysis revealed that the six most important factors are the percentage of trucks, traffic volume, slope length, road grade, curvature, and curve length, with the feature contribution degree of 16.9%, 14.4%, 12.5%, 12.1%, 7.7%, and 7.6%, respectively. All these factors are related to traffic and road conditions, indicating their significant impact on traffic accident severity.
2. Focusing on the six most important factors, we visualized the relationships between single and dual factors and the three classes of traffic accident severity by drawing the partial dependence plot. The results show that there is an obvious nonlinear relationship and threshold effect on accident severity.
3. Concentrating on traffic accidents with injuries and fatalities (WIWF), we conducted a detailed analysis of the threshold and interaction effects of two factor related to traffic and road features. The results highlight specific traffic statuses, road geometry features, and their combinations, such as the curve and slope combinations and the small-radius curves, especially under traffic conditions where the truck ratio rangs from 10% to 20% and the traffic volume rangs from 20,000 to 30,000 pcu/day. These features significantly contribute to WIWF accidents. Therefore, safety management should be strengthened on these road segments by establishing necessary warning and protection facilities, and taking relevant actions to control the traffic volume and the proportion of trucks.

The findings drawn from this study can inform highway design and safety management. However, there are several limitations. Firstly, the accident data utilized in this paper were sourced from a specific highway section over a one-year period, which may constrain the generalizability and applicability of the findings to other contexts. The current work should be extended to gathering more reliable data sources for further examination. Secondly, while vehicle-related and driver-related factors, particularly those associated with driver’ age, gender, and unsafe driving behavior factors, are known to significantly influence accident severity, these factors were not included in our analysis due to data availability constraints. Therefore, future research may focus on collecting more comprehensive data to facilitate a deeper exploration of the complex interactions and nonlinear effects of various factors on traffic accidents, ultimately providing more valuable insights for the highway safety management.

## Supporting information

S1 File The dataset used in this study. (XLSX)

S2 File The partial dependence plots of undiscussed factors in the main text. (DOCX)
