# Supplementary material for: Nonlinear effects of traffic statuses and road geometries on highway traffic accident severity: A machine learning approach
Source: PLoS One. 2024 Nov 22;19(11):e0314133. doi: 10.1371/journal.pone.0314133 (PMC11584126; doi:10.1371/journal.pone.0314133)
Supplement: S2 File — (DOCX) [file pone.0314133.s002.docx]

**S2. The partial dependence plots of undiscussed factors in the main text**

Our paper, “Nonlinear effects of traffic statuses and road geometries on highway traffic accident severity: a machine learning approach”, focuses on the nonlinear effects of traffic status and road geometry on the severity of road accidents, employing a random forest model to construct this nonlinear relationship. In the model, we considered 14 variables, including combined alignment, horizontal alignment, tangent length, curve length, curvature, superelevation, road grade, slope length, traffic volume, percentage of trucks, weather conditions, lighting conditions, and day type. In the main text, we analyzed the nonlinear effects of the six most influential factors—percentage of trucks, daily traffic volume, slope length, road grade, curve radius, and curve length—on accident severity using partial dependence plots, as well as their partial interactions. Other single factors not considered in the main text, along with interactions involving these six key factors, are presented here, as seen in Fig A1, and Fig A2.


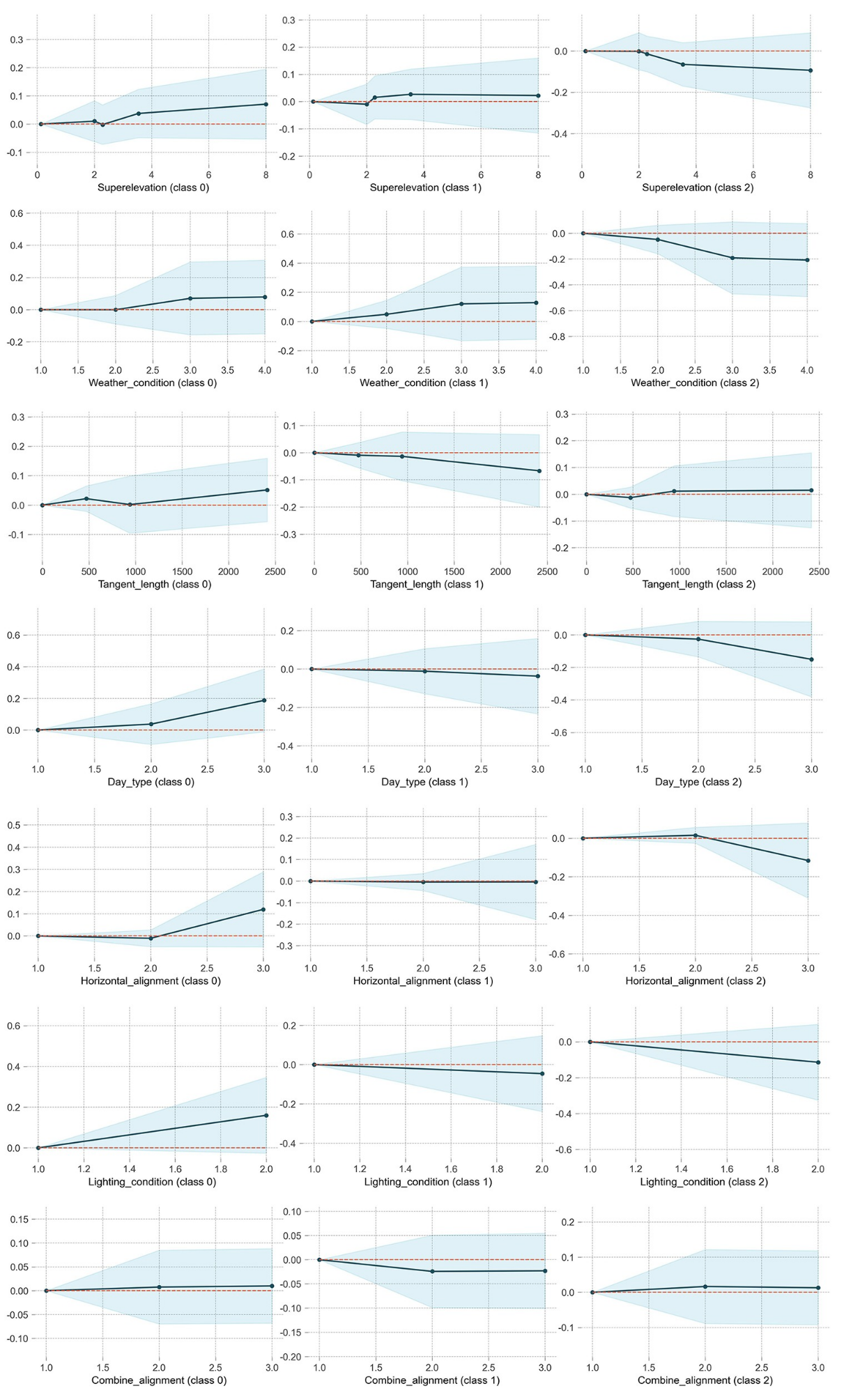


Fig A1. The partial dependence plot of super elevation, weather condition, tangent length, day type, horizontal alignment, lighting condition, and combine alignment


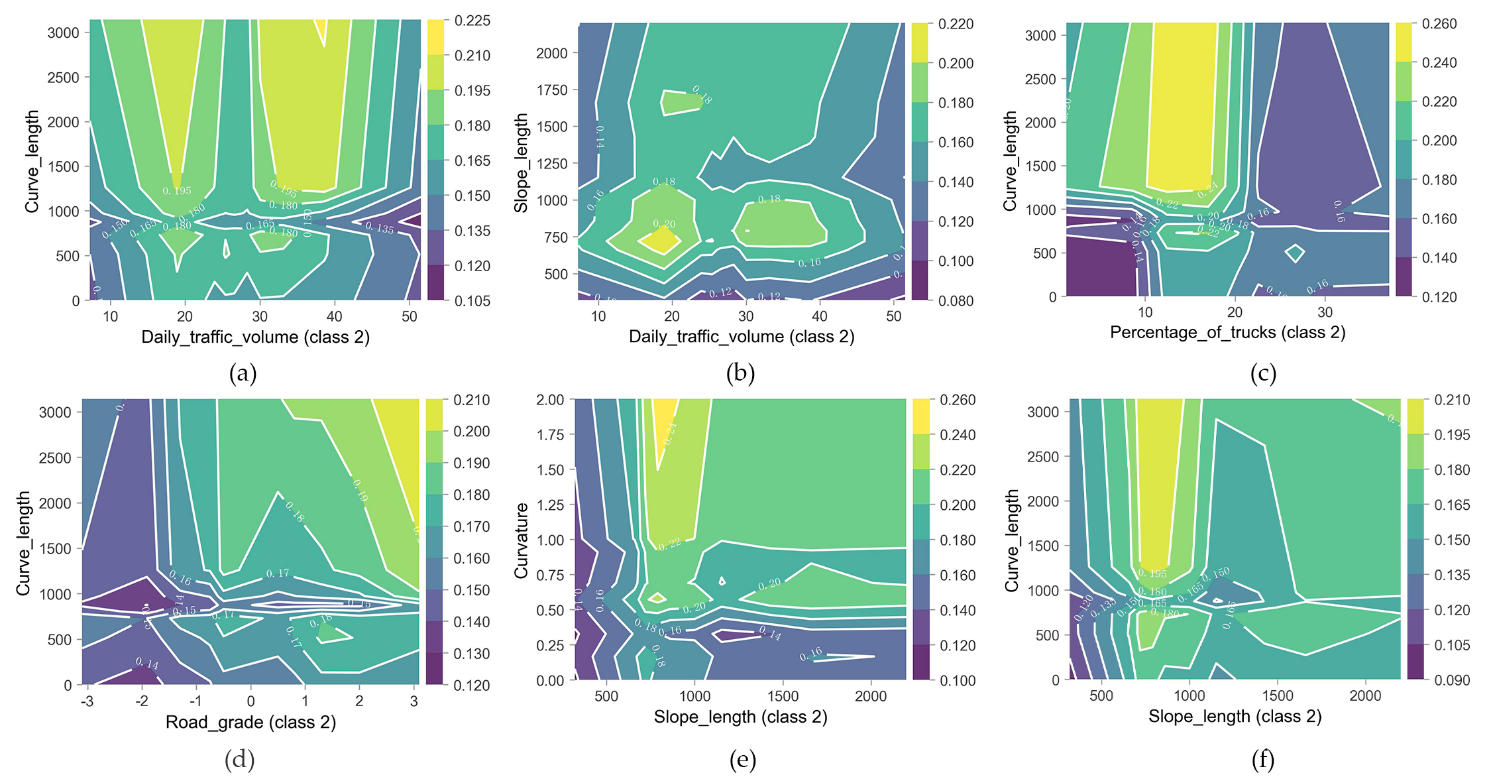


Fig A2. The partial dependence plots for WIWF accident under traffic status and road geometry factors other than those covered in Fig 10 of the main text
